# Provision of cervical cancer prevention services in Northern Uganda: a survey of health workers from rural health centres

**DOI:** 10.1186/s12913-021-06795-5

**Published:** 2021-08-11

**Authors:** James Henry Obol, Sophia Lin, Mark James Obwolo, Reema Harrison, Robyn Richmond

**Affiliations:** 1 School of Population Health and Community Medicine, University of New South Wales, 2033 Kensington, NSW Australia; 2 Faculty of Medicine, Gulu University, P.O Box 166, Gulu, Uganda

**Keywords:** Cervical cancer, Health worker, Prevention services, Northern Uganda

## Abstract

**Background:**

Cervical cancer is the leading cancer among Ugandan women, contributing to 40 % of all cancer cases recorded in the cancer registry. Having identified the substantial impact of cervical cancer among Ugandan women, the Ministry of Health in 2010 launched a Strategic Plan for Cervical Cancer prevention and control. This study was conducted to determine if health workers working in rural health centres (HCs) III and IV in Northern Uganda provide cervical cancer screening services as recommended in the Strategic Plan.

**Methods:**

A cross-sectional survey using a structured questionnaire was conducted among nurses, midwives and clinical officers working in rural HC III and IV in Northern Uganda. Data were entered in Epidata 3.1 and analysed using Stata 16 statistical software. Univariate, bivariate, and multivariate analyses were performed. Any factor with *p*-value ≤ 0.05 was considered a significant predictor of outcome.

**Results:**

We surveyed 286 health workers. Fifty-one (18 %) health workers were screening women for cervical cancer. Fifty-eight (21 %) health workers have guideline for cervical cancer screening in their HCs, 93 (33 %) participants were trained to screen women for cervical cancer. Two hundred sixty-two (92 %) participants provided HPV vaccination. Two hundred forty-six (87 %) participants were conducting health education about cervical cancer in their HCs. Factors associated with screening women for cervical cancer include: being a staff member from HCs III (AOR = 0.30, 95 % CI 0.13–0.68, *p* = 0.00), being staff of HCs that have organization to support cervical cancer screening services (AOR = 4.38, 95 % CI 1.99–9.63, *p*-=0.00), being a health worker who had been trained to screen for cervical cancer (AOR = 2.21, 95 % CI 1.00–4.90, *p* = 0.05) and staff from HCs that has guideline for cervical cancer screening (AOR = 2.89, 95 % CI 1.22–6.86, *p* = 0.02).

**Conclusions:**

This study shows an overall structural problem related to the delivery of cervical cancer screening services in HC III and IV in Northern Uganda which the Strategic Plan has not addressed. These structural problems need urgent attention if the Uganda government and other sub-Saharan African (SSA) countries are to achieve the World Health Organization (WHO) 90–70–90 targets by 2030 to be on track for cervical cancer elimination.

**Supplementary Information:**

The online version contains supplementary material available at 10.1186/s12913-021-06795-5.

## Background

Cervical cancer is a preventable disease and yet many women continue to be undiagnosed and subsequently die of the disease each year not only because of problems with cervical cancer prevention but also because of problems with early detection and control [1, 2]. Globally, cervical cancer incidence and mortality is disproportionately distributed with the highest burden falling among low-and-middle income countries (LMICs) of the world, particularly in regions such as sub-Saharan Africa (SSA), Southern and South-Eastern Asia, South America and the Caribbean [2–4]. About 85 % of cervical cancer incidence and mortality globally occurs in LMICs [5] and this represent a failure in healthcare systems to implement an efficient strategy for cervical cancer prevention and control [6].

Early screening for cervical precancerous lesions with low cost methods such as visual inspection with acetic acid (VIA) can detect cervical intraepithelial neoplasia (CIN) grade 2 or 3 which are cervical precancerous lesions [7–11]. The cervical precancerous lesions can be treated with cryotherapy which is a simple but effective treatment method to halt the development of CIN into cervical cancer so that the woman can live a healthy life [7, 8, 10, 11]. Therefore, for cervical cancer screening to be beneficial, all women who screened positive for CIN need to receive prompt and effective treatment for the CIN detected [7, 11, 12].

Developed countries have initiated and sustained cervical cancer screening programmes among sexually active women with high coverage resulting in reduction in rates of cervical cancer [12, 13]. However, uptake of cervical cancer screening has remained low in LMICs [14, 15]. Most LMICs suffer from limited resources, a weak or fragmented health system which contributes to the high burden of cervical cancer [1]. Additionally, in most SSA countries there is a lack of trained staff to conduct cervical cancer screening and deliver treatment, and availability of infrastructures to aid cervical cancer screening and treatment [15–18]. All the above factors have affected initiation or sustainability of cervical cancer screening and treatment programme [12, 15, 16]. Among the few SSA countries that have initiated a cervical cancer screening programme, the proportion of women accessing screening services has remain very minimal [15, 16, 18–20] and inconsistent due to lack of resources or financial commitment from government [15, 17]. The World Health Organization (WHO) estimate that about 90 % of cervical cancer deaths are due to poor access to prevention, screening, and treatment [21].

In Uganda, cervical cancer has been reported as the leading cancer affecting women [22, 23] with annual incidence of 6,413 cases and annual mortality of 4,301 deaths [23]. Cervical cancer is responsible for up to 40 % of all cancers reported in Uganda [18].

Organised population based cervical cancer screening has helped to reduced cervical cancer incidence and mortality in developed countries [13]. However, in Uganda, there is lack of organised population based cervical cancer screening programme resulting into minimal access to cervical cancer screening services [18]. This has led to low uptake of cervical cancer screening in Eastern Uganda (4.8 %) [24] and Central Uganda (7 %) [25] as well as in population sub-groups: 30.3 % among Human immunodeficiency virus (HIV) positive women [26] and 19 % among female health workers [27].

The Ugandan health system is decentralised, divided into national and district health systems [28, 29]. Overall, the Ministry of Health is responsible for making policy, planning, budgeting, liaising with external bodies such as WHO, setting regulations and standard of healthcare, and allocating funds to district healthcare systems [29, 30]. The district health system is under the district local government [31] and managed by a District Health Officer (DHO) [29]. The DHO is responsible for mobilisation and deployment of both human and non-human resources, such as medical supplies and equipment, to the hospitals and health centres (HCs) as well as supervision of healthcare delivery within those health facilities [30, 32]. Structurally, the district health system has the village health teams (VHTs) as the lowest level of healthcare delivery regarded as HC level I (HC I) [29, 30, 33]. This is then followed by HC II – IV and then district hospital which is the highest level of healthcare within a district [34]. The healthcare packages provided becomes larger at each level of healthcare delivery as the population also increases in size for the services [30].

To address the challenges of cervical cancer screening programme in Uganda, the Uganda Ministry of Health developed and rolled out the national Strategic Plan for Cervical Cancer Prevention and Control guideline (2010–2014) [18]. The Strategic Plan has six priority areas but emphasis was on prevention of HPV infection through vaccination of girls aged 10 to 14 years, low cost screening using visual inspection with acetic acid (VIA), and treatment of CIN using cryotherapy for women aged 25 to 49 years [18]. The Strategic Plan targeted at least 80 % screening and treatment coverage for women aged 25 to 49 years with CIN by 2015. The Strategic Plan adopted screening interval of three years for HIV negative women and annually for HIV positive women [18]. To achieve these targets, the Strategic Plan aimed to train 80 % of operational-level health workers by 2015 to perform cervical cancer screening and treat CIN [18]. In HCs IV, at least six health workers were to be trained while in HCs III, three health workers were to be trained to implement cervical cancer screening and treatment services. The Strategic Plan has set national rollout of screening and treatment by 2013 for HCs IV, and HCs III was to start nationwide cervical cancer screening and referral of women found with CIN for treatment by 2014 [18].

Screening for CIN using VIA and treatment with cryotherapy can be performed by nurses, midwives and clinical officers [17, 18, 35, 36]. In Uganda, nurses, midwives and clinical officers form most of health workers in rural area where the majority of the population resides [37]. Under the Strategic Plan, nurses, midwives and clinical officers would provide screening for cervical cancer using VIA and treat CIN using cryotherapy [18]. This would provide access to vital preventive services in communities that would otherwise have no access to cervical cancer screening and treatment services. However, there is no information on the implementation of this Strategic Plan nor the uptake of cervical cancer screening which has remained low as indicated in several studies conducted in different regions of Uganda [24–26, 38]. This study aims to determine if nurses, midwives, and clinical officers working in rural HCs are providing cervical cancer screening services as recommended by the Strategic Plan.

## Methods

### Study design and setting

This study uses a cross-sectional survey design. The study was conducted among health workers in rural HC III and IV in eight districts in Acholi sub-region, Northern Uganda. At the time of the survey, the region had 64 HCs III and 8 HCs IV [39]. According to the Strategic Plan, a HC III is expected to conduct health education about cervical cancer, conduct HPV vaccination of girls aged 10 to 14 years and perform cervical cancer screening using VIA. Women with cervical precancerous lesions are then referred to HC IV or to the district hospital for treatment using cryotherapy [18]. The Strategic Plan stipulates that a HC IV will conduct health education about cervical cancer, conduct HPV vaccination of girls aged 10 to 14 years and provide women with cervical cancer screening using VIA and treat cervical precancerous lesions using cryotherapy [18]. The research participants were distributed in HC III and IV as in the Table 1 below.

**Table 1:** Research participants’ distribution according to HC level

Health workers’ qualifications	Numbers in HCs III	Numbers in HCs IV	Total
Clinical Officers	39	13	52
Midwives	65	16	81
Nurses	118	35	153
Total	222	64	286

### Sample and selection criteria

We purposively sampled nurses, midwives and clinical officers working in rural HCs III and IV in the eight districts of Acholi sub-region of Northern Uganda. Participants were included if they were employed for at least one year. For nurses and midwives, they must have been registered with the Uganda Nurses and Midwives Council while clinical officers must have been registered with the Allied Health Professional Council of Uganda. We excluded health workers who were on study leave which is granted to an employee to enable him/her to have sufficient time to study. This person would then not be available at the workstation for the period of the leave granted. Excluding them would not compromise our research validity as health workers are granted study leave only when there are sufficient health workers to replace them in the HC.

### Sample size estimation

A minimum required sample size of 271 participants was estimated using the modified Kish Leslie formula of 1965 [40]. The desired level of confidence was set at 90 % confidence interval (CI). The proportion of the health workers conducting cervical cancer screening was set at 0.5 since it was unknown; and the level of precision desired was set at 0.05. We increased the sample size by 5 % to account for non-response and withdrawal of consent from participation in the survey giving us a total sample of 285 participants.

### Sampling HCs

All 8 HC IV were included in the survey and 54 out of 64 HC III [39] were randomly sampled. We chose 54 HC III because we aimed to survey four participants from each HC III and eight participants from each HC IV to ensure equal representation in the survey. This would amount to sampling of 216 health workers from HC III and 64 health workers from HC IV. Adding the numbers of samples from HC III and HC IV will total to 280 participants.

To generate the list of 54 HC III, the names of the HC III were written alphabetically. They were then given a number from 1 to 64 corresponding to each name in alphabetical order. We then used software [41] to generate 54 random numbers between 1 and 64. These random numbers were then matched with each of the numbers for the names of HCs to enable us to select all the 54 HC III.

### Study participants recruitment

After generating the names of HCs for the survey, the principal investigator (PI) visited each HC and discussed the survey of health workers with the head of the HC who gave a verbal clearance to conduct the study. Clearance was made smoother as PI had obtained administrative clearance from the office of the Chief Administrative Officer who is the overall head of public service at the district level [42]. The study was advertised in each of the sampled HCs for two reasons: First, to create awareness about the study among health workers in each HC. Secondly, to allow health workers who would be away (absent or on leave), but who were interested in participating in the study, to contact the PI so that PI could brief them about the study before conducting the survey. In the districts and HCs where we conducted the research, we could not apply simple random sampling because staff registers were incomplete or absent. However, we visited each HC three times on different occasions so that staff who were not available during our previous visit might be available so that we could conduct the survey. In each of the visits we made to the health centres, participants were consecutively sampled from their workstation for participation in the study until we had accrued the required sample size.

### Data collection tool and process

A self-administered structured questionnaire was used to obtained data on: Socio-demographic and HC characteristics of participants such as age; sex; number of years employed as a health worker; participant’s qualifications; participants’ workplace (HC level); HC partnerships with non-governmental organizations (NGOs) and other agencies; HC having any guidelines for cervical cancer screening; and district in which the HC is located. The questionnaire also asked questions whether participants have ever been trained to conduct cervical cancer screening. The questionnaire also sought information about cervical cancer screening services in the HC; reasons for not screening women for cervical cancer, cervical cancer screening methods; how often HC screen women for cervical cancer at the HC; provision of counselling services before a woman undergo cervical cancer screening; community members being aware of cervical cancer screening services offered at the HC; services provided to women screened and found with cervical precancerous lesions; HC receive support supervision for cervical cancer screening services provided. In addition, we sought information on whether the HC conduct HPV vaccination of girls; whether community is aware of the HPV vaccination service at the HC; participant conduct health education about cervical cancer at the HC; participant conduct outreach health education about cervical cancer in the community; and reasons for not conducting outreach health education about cervical cancer in the community; participant’s awareness of Strategic Plan for Cervical Cancer Prevention and Control in Uganda; participant’s awareness of age group for cervical screening using VIA as recommended by Strategic Plan.

The last part of the questionnaire asked participants who were trained whether they feel competent to screen women for cervical cancer. We also asked if participants were willing to be trained on methods of screening for cervical cancer using VIA methods. We sought information about willingness to initiate cervical cancer screening in the HC; and reasons why the participants were not willing to start cervical cancer screening in their HCs. The questionnaire developed for this study is provided as Additional File 1. Participants took approximately 30 min to complete the questionnaire, and each was given a bar of soap (approximately US$0.54) as compensation for their time during the survey.

### Operational definition of outcome measure

Our outcome measure was “screening women for cervical cancer by health workers” which was measured by either “yes” if the health worker was screening women for cervical cancer or “no” if the health worker was not screening women for cervical cancer.

### Quality control

Two research assistants were trained by PI for two days on the data collection tool to ensure quality data collection, consent procedure, confidentiality, and privacy. The PI and research assistants pre-tested the survey questionnaires among 15 nurses, midwives, and clinical officers who were working in three HCs III within Gulu Municipality. This helped us identify key issues that may affect response to the questionnaire such as meaning of certain terms, and phrasing of questions, so that they could be corrected in the revised questionnaire. The PI together with the research assistants collected data from research participants. In addition, the PI supervised data collection process to ensure that the questionnaires were completed and handed over immediately. Data were entered two times and merged and cleaned to minimise error in data entry.

### Data management and analysis

The PI used Epidata version 3.1 [43] to create the database for data entry. Data was exported to Stata version 16 [44] statistical software for analysis. Univariate analysis was performed and displayed in tables. Continuous variables were categorised using means. Chi-square test or Fisher’s exact test was used for sub-group analysis. We reported Odds Ratio (OR) and 95 % confidence interval (CI) and any factor with *p*-value ≤ 0.05 was reported as significant at the bivariate level. Variables with *p*-value ≤ 0.2 in the bivariate analysis were fitted in the multivariate model. The variable health workers’ districts were omitted from the logistic regression model as one cell had zero value. The variable sex of health workers was retained in the final model as it is a potential covariate which could affect cervical cancer screening practice of the health worker despite having a *p*-value > 0.2 at the bivariate analysis. The variables entered in the multivariate model were simultaneously analysed using logistic regression to control the effect of each variable in the model. Variables with *p*-value ≤ 0.05 were taken as a significant predictor of screening women for cervical cancer by the health workers.

### Ethical considerations

The study was approved by University of New South Wales School of Public Health and Community Medicine Human Research Ethics Committee (HC180508). The study was approved by Gulu University Research Ethics Committee (GUREC-090-18) and registered with Uganda National Council for Science and Technology with registration number SS4839. Administrative clearances to conduct the study were granted by Chief Administrative Officer from each of the eight Districts Local Governments which form Acholi sub-region. All research participants provided written informed consent before participating in the survey. The information obtained during the study is being treated with utmost strict confidentiality.

## Results

### Study population

A total of 286 health workers working in HCs III and IV in Northern Uganda participated in the survey. Ten health workers declined to participate in the study expressing disinterest. Five participants did not return their questionnaire when they were called to a medical emergency or delivery. We made several attempts to retrieve questionnaires, but our efforts were futile due to annual leave, or off duty or absence from their workstation.

### Demographic and health centre characteristics

There were 222 (78 %) health workers working in HCs III and majority of participants were nurses 153 (54 %). 167 (60 %) were aged between 21 and 35 years. One hundred eighty-eight (66 %) participants were female. One hundred ninety-two (68 %) participants had worked between 1 and 10 years. Ninety-three (33 %) participants were trained to screen women for cervical cancer. Fifty-eight (21 %) health workers indicated they have cervical cancer screening guideline in their HCs. Table 2 summarises the demographic and HC characteristics.

**Table 2:** Demographic and health centre characteristics of health workers surveyed (*N* = 286)

Variables	Frequency (n)	Percentage (%)
**Health workers’ health centre level** (***n*** = **286**)		
Health centre level III	222	78.0
Health centre level IV	64	22.0
**Age groups of health workers** (***n*** = **280**)		
21–35	167	60.0
36–57	113	40.0
**Sex of health workers** (***n*** = **286**)		
Females	188	66.0
Males	98	34.0
**Qualifications of health workers** (***n*** = **286**)		
Nurse	153	54.0
Midwife	81	28.0
Clinical officer	52	18.0
**Numbers of years working** (***n*** = **281**)		
1–10 years	192	68.0
11–38 years	89	32.0
**Health workers trained to screen for cervical cancer** (***n*** = **283**)		
No	190	67.0
Yes	93	33.0
**HC has an Organisation that support cervical cancer screening** (***n* = 274**)		
No	170	62.0
Yes	104	38.0
**HC has guidelines for cervical cancer screening** (***n*** = **281**)		
No	223	79.0
Yes	58	21.0
**Health workers’ Districts**		
Agago	28	10.0
Amuru	49	17.0
Gulu	33	12.0
Kitgum	30	10.5
Lamwo	38	13.0
Nwoya	15	5.0
Omoro	30	10.5
Pader	63	22.0

### Provision of cervical cancer screening services

There were 51 (18 %) health workers who reported screening women for cervical cancer in their HCs. Fifty-two (57 %) participants indicated that they rely on outreach from other organizations to conduct cervical cancer screening. Reasons cited for not screening women for cervical cancer includes: No equipment or consumable, 183 (35 %) participants; Lack of skills because no one was trained to screen for cervical cancer, 172 (33 %) participants; lack of personnel, 86 (16 %) participants. Forty-nine (98 %) of health workers who screen women for cervical cancer indicated counselling women before conducting screening. Table 3 describe cervical cancer prevention services provided by health workers from HCs III & IV.

**Table 3:** Cervical cancer prevention services provided by health workers from HC III & IV

Variables	Frequency (n)	Percentage (%)
**Health workers screening women for cervical cancer in the health facility*n*** = **286**		
Yes	51	18.0
**Reasons for not screening women for cervical cancer (** ***Multiple responses)***		
No equipment/consumables	183	35.0
Lack of personnel	86	16.0
No space/room for screening	50	10.0
Lack skill because no one is trained to conduct screening	172	33.0
No time for screening because of heavy workload	18	3.0
Screening increases my workload	3	1.0
Others	13	2.0
**Cervical cancer screening methods (Multiple responses allowed)**		
VIA	37	76.0
Pap smears	7	14.0
Liquid-based cytology	4	8.0
HPV DNA testing	1	2.0
**How often do you screen women for cervical cancer in this health facility?*n*** = **92**		
1–3 days in a week	25	27.0
4–7 days in a week	15	16.0
Screening is through outreach by other organisations	52	57.0
**Counselling women before screening for cervical cancer?*n*** = **50**		
Yes	49	98.0
**Community awareness that the health facility offers cervical cancer screening.*n*** = **54**		
Yes	50	93.0
**Procedure offered to a woman found with a precancerous lesion.*n*** = **82**		
Referred for treatment to next level of care	77	94.0
Treatment offered at this health centre	4	5.0
Inform her but do not refer or treat	1	1.0
**Support supervision for cervical cancer prevention services offered in this health facility.*n*** = **78**		
Yes	33	42.0
**HPV vaccination of adolescent girls offered in this health facility.*n*** = **285**		
Yes	261	92.0
**Community members aware of HPV vaccination offered at this health facility. *n*** = **261**		
Yes	244	93.0
**Health education on cervical cancer offered at the health facility.*n*** = **283**		
Yes	246	87.0
**Reasons for not conducting health education in the health facility. (Multiple responses allowed)**		
No health education material	27	44.2
Cervical cancer not a priority disease here	10	16.4
Government is not promoting	10	16.4
Other reasons^a^	14	23.0
**Conducting outreach health education on cervical cancer in the community.*n*** = **283**		
Yes	128	45.0
**Reasons for not conducting outreach health education in the community. (Multiple responses)**		
No transport	88	29.0
No fund	90	30.0
No staff	8	3.0
No time because of heavy workload	8	3.0
Not trained on cervical cancer	82	27.0
Others	27	9.0
**Health workers’ aware of age group for cervical cancer screening using VIA** (***n*** = **279**)		
Yes	171	61.0

Other reasons^a^ (lack of knowledge and skills, inadequate staffing, reluctant and cervical cancer not common)

### Attitudes

**Fig. 1:**
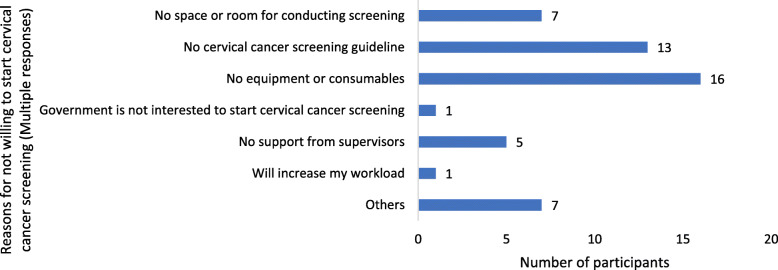
Reasons for not willing to start cervical cancer screening by 21 participants in their HCs

Among participants who were trained, 40 (43 %) indicated they feel incompetent to screen women for cervical cancer. Among all participants, 270 (96 %) were willing to undergo training on how to screen women for cervical cancer using VIA. There were 253 (92 %) participants who were willing to start cervical cancer screening in their HCs. Only 21 (8 %) participants were not willing to start cervical cancer screening in their HCs. Figure 1 summarises the reasons provided by the participants who were not willing to start cervical cancer screening in their HCs.

### Demographic and HCs characteristics as predictors of screening women for cervical cancer

The following factors were significant predictors that health workers would screen women for cervical cancer: Being health workers from HCs III (AOR = 0.30, 0.13–0.68, *p* = 0.00), health workers trained to conduct cervical cancer screening (AOR = 2.21, 95 % CI 1.00–4.90, *p* = 0.05), being health workers from HCs having an organization that supported cervical cancer screening (AOR = 4.38, 95 % CI 1.99–9.63, *p* = 0.00), and health workers from HCs with guideline for cervical cancer screening (AOR = 2.89, 95 % CI 1.22–6.86, *p* = 0.02). Table 4 summarises the results of the bivariate and multivariate analysis of factors associated with screening women for cervical cancer by health workers.

**Table 4:** Bivariate and Multivariate results of factors associated with conducting cervical cancer screening by health workers from rural HC III & IV, northern Uganda. *N* = 286

	Total, *N* = 286	COR	95 % CI	*p*-value	AOR	95 % CI	*p*-value
**Variables**							
** Health workers’ health centre level** (***n*** = **286**)							
Health centre level IV	64 (22)	1			1		
Health centre level III	222 (78)	0.23	0.12–0.44	0.00	**0.30**	**0.13–0.68**	**0.00**
** Age groups of health workers** (***n*** = **280**)							
21–35	167 (60)	1			1		
36–57	113 (40)	2.54	1.35–4.76	0.00	1.05	0.34–3.23	0.94
** Sex of health workers** (***n*** = **286**)							
Females	188(66)	1			1		
Males	98 (34)	1.3	0.70–2.42	0.41	1.03	0.40–2.65	0.96
** Qualifications of health workers** (***n*** = **286**)							
Nurse	153 (54)	1			1		
Midwife	81 (28)	0.89	0.42–1.88	0.76	1.13	0.42–3.04	0.80
Clinical officer	52 (18)	1.89	0.89–3.98	0.10	1.66	0.59–4.65	0.33
** Numbers of years working** (***n*** = **281**)							
1–10 years	192 (68)	1			1		
11–38 years	89 (32)	2.76	1.48–5.13	0.00	2.27	0.72–7.14	0.16
** Health workers trained to screen for cervical cancer** (***n*** = **283**)							
No	190 (67)	1			1		
Yes	93 (33)	2.68	1.44–5.00	0.00	**2.21**	**1.00 -4.90**	**0.05**
** HC has an Organisation that support cervical cancer screening** (***n*** = **274**)							
No	170 (62)	1			1		
Yes	104 (38)	4.59	2.34–9.01	0.00	**4.38**	**1.99–9.63**	**0.00**
** HC has guidelines for cervical cancer screening** (***n*** = **281**)							
No	223 (79)	1			1		
Yes	58 (21)	4.26	2.20–8.25	0.00	**2.89**	**1.22–6.86**	**0.02**
** Health workers’ aware of age group for cervical cancer screening using VIA** (***n*** = **279**)							
No	108 (39)	1			1		
Yes	171 (61)	2.29	1.13–4.60	0.02	1.57	0.60–4.08	0.36

Bolded figures indicate statistically significant variables at *p* ≤ 0.05 at multivariate analysis. Our final model was checked using Hosmer-Lemeshow goodness-of-fit test to see whether it fit perfectly well. The *p*-value of 0.99 from the Hosmer-Lemeshow’s goodness-of-fit test is an indicator that our model fitted the data well

*COR* Crude Odds Ratio, *AOR *Adjusted Odds Ratio

## Discussion

This study revealed that less than one fifth of the surveyed health workers screen women for cervical cancer in their HCs. However, this information fall short of the Strategic Plan target of ensuring that all HCs III and IV will provide cervical cancer screening throughout Uganda by 2014 [18]. Even among participants who indicated that screening women for cervical cancer occurs in their HCs, a greater proportion relied on other organizations to come and screen women for cervical cancer during health camp outreaches in their HCs. Among participants who were screening women for cervical cancer, most of them stated that they counsel women before screening for cervical cancer. Alleviating anxiety among women is important and women should be provided with information which make them understand that cervical cancer screening is not a test for cancer [7]. Participants from a study conducted in Nigeria also stressed the importance of counselling women before screening women for cervical cancer [45].

Majority of the participants cited the lack of equipment and reagent as one of the major reasons for not conducting cervical cancer screening in their HCs. An earlier study in Uganda in 2006 revealed that most health workers cited the lack of vaginal specula as the reason for not screening women for cervical cancer [27]. The lack of equipment and reagents have persisted as a major barrier in the provision of cervical cancer screening for Ugandan women despite the Strategic Plan which articulates the need for provision of supplies to the HCs [18]. Multiple studies conducted within SSA countries such as Nigeria, Malawi, Tanzania, Ethiopia and Kenya have cited the lack of equipment and supplies as one of the major barriers to the provision of cervical cancer screening services [16, 45–49].

Our study found that only 33 % of the participants had ever been trained to screen for cervical cancer contrary to the Strategic Plan target of 80 % by 2015 [18]. Health workers who indicated that they were trained to screen for cervical cancer were more likely to provide cervical cancer screening services in their HCs than health workers who were not trained. Lack of training was one of the most common reasons cited by participants for not conducting cervical cancer screening and outreach health education in the community. However, among the participants who indicated to have been trained, 43 % felt they were incompetent to screen women for cervical cancer. An earlier study conducted in Uganda found the need for health training curricula review to incorporate practical skill of cervical cancer screening [27]. Lack of well-trained health workers as barrier to the provision of cervical cancer screening services have been reported in many SSA countries such as in Malawi, Ethiopia, Tanzania, Nigeria, Kenya, and South Africa [16, 45, 46, 48–50].

Our study found that health workers from HCs III were 70 % less likely to screen women for cervical cancer than health workers from HCs IV. During our survey we noted a general understaffing in most HCs III in Northern Uganda which is contrary to the staffing level recommended by the Uganda Ministry of Health [30, 51]. Our participants cited the lack of personnel as the reason for not providing cervical cancer screening at the HCs. An earlier report by the Uganda Ministry of Health indicates that nearly half of the districts in Uganda were operating below 50 % of the approved staffing level [52]. Many studies conducted within SSA have reported inadequate numbers of personnel as one of the major barrier to provision of cervical cancer screening [16, 49, 50]. Furthermore, we observed when we attended the HCs for interviews, most Heads of the HCs III who should have been supervising service delivery were absent. In some HCs, we made repeated visits to the HCs and on three occasions we could not find all the staff within. In some HCs we found only nursing assistants attending to patients and not nurses themselves. Multiple studies have documented absenteeism among health workers in the Uganda public health sector [53–55]. The Uganda Ministry of Health estimate absenteeism rates among health workers in public health facilities to range from 47 to 50 % [52]. The factors described above do not favour the provision of cervical cancer screening within HC III settings.

Participants who indicated to have organizations that support cervical cancer screening in their HCs were more likely to screen for cervical cancer than staff from HCs without other organizational support. In Northern Uganda, there are few NGOs such as Reproductive Health Uganda, Marie Stopes Uganda, and the Gulu University – University of New South Wales (GU-UNSW) collaboration which are providing cervical cancer screening services through local HCs. These organisations have acetic acid which is not provided to HCs as it is regarded as a second line item by Uganda Ministry of Health [56]. However, the Strategic Plan adopted the VIA as the screening methods of choice [18] and the lack of acetic acid undermine cervical cancer screening effort in Uganda. Furthermore, when we stratify our outcome by the HCs whose staff received training and clinical consumables from GU-UNSW, the finding was statistically significant (χ^2^ = 5.88, *p* = 0.02). This underscores the need for the HCs in this region to develop partnership with universities and other NGOs to help fill the gap created by government inability to provide basic items essential for cervical cancer screening due to limited resources.

In our study, a greater proportion of the health workers were providing vaccination of young girls against HPV in their HCs. Since 2015 the global alliance for vaccine and immunisation (GAVI) has funded HPV vaccinations in Uganda and HPV vaccination has been integrated with other childhood vaccinations that the HCs conduct [57, 58]. This further explains why most of the health workers reported that the people in their community were aware of the HCs conducting immunisation of young girls against HPV.

A greater proportion of the health workers indicated that they provide health education about cervical cancer in their HCs. However, less than half of the participants indicated they go for outreach health education on cervical cancer in the community. Reasons cited were lack of funds for their allowances during the outreach, lack of transport for travelling to the community and lack of training in cervical cancer. This has affected the extent to which young girls aged 10–14 years are immunised in Uganda against HPV causing a drop in uptake of HPV second dose which is at 41 % [59] against national target of 80 % [18].

There were 97 % of the participants who indicated that their HCs do not receive funding to support cervical cancer activities. Funding cervical cancer activities at the HCs level will ensure that health workers can afford transport and have allowances while in the community. Addressing these gaps by district local governments and Uganda Ministry of Health will improve delivery of cervical cancer services in the community. More importantly, this would ensure that women would be reached for provision of screening services.

Our study found that health workers who indicated that their HCs have guideline for cervical cancer screening were more likely to screen for cervical cancer than staff from HCs without guideline for cervical cancer screening. In Uganda, cervical cancer screening guideline is useful for guiding health workers with information such as the age group for screening, screening methods and frequency of cervical cancer screening [18]. A study conducted in Malawi found that most providers of cervical cancer prevention services were not aware of the guidelines for cervical cancer screening and treatment [16]. In our study, about 25 % of the participants stated that they were aware of the Strategic Plan for Cervical Cancer Prevention and Control in Uganda. However, only 2 % of the health workers knew the period covered by the Strategic Plan for Cervical Cancer Prevention and Control in Uganda. This means since the launched of the Strategic Plan in 2010, there has been little effort to disseminate the Strategic Plan to health workers to implement. This calls for urgent need by the Uganda Ministry of Health to provide HCs with copies and conduct orientation of health workers who will be implementing the Strategic Plan.

Our study indicates that there was no association between screening women for cervical cancer and staff qualifications. We explored this as it would have been expected that in this setting, midwifery qualification would make someone more likely to understand female reproductive system. Furthermore, their work environment is about provision of reproductive health services to women than the nurses or clinical officers.

In this study it is worthy to note that majority (96 %) of the participants were willing to be trained on how to screen women for cervical cancer using VIA methods as prescribed by the Strategic Plan. Furthermore, most (92 %) of the participants who were not screening women for cervical cancer in their HCs were willing to start cervical cancer screening in their HCs. The positive attitudes exhibited by the health workers is important for the introduction of the cervical cancer screening in HCs across Northern Uganda. Studies conducted within SSA found that the lack of willingness and or commitment by health workers was a barrier in the implementation of cervical cancer screening programme [16, 50].

### Strengths and limitations

Most studies on cervical cancer in Uganda have focussed mainly on uptake of cervical cancer screening by women and have ignored the health workers who provide the screening services. Our study is the first study which has examined provision of cervical cancer screening services by health workers working in rural HCs in Uganda based on the Strategic Plan. This is important because, if health workers are not providing the screening services, then Uganda Ministry of Health cannot reverse the worrying trend of cervical cancer in the country. Findings from this study will help shed light on the implementation of the Strategic Plan by rural health workers in Uganda. This is because most population and HCs are in rural areas of Uganda in which rural women seek care first. The findings will help guide developing an effective cervical cancer control program especially targeting health workers and healthcare infrastructures in Uganda and other SSA countries.

The study findings must be considered in the context of its limitations; social desirability bias may have influenced more health workers to respond that they were providing cervical cancer screening services than the actual numbers of participants who were screening women for cervical cancer. This study was conducted among nurses, midwives and clinical officers working in rural HC IV and III which are under district local governments. Therefore, the findings cannot be generalised to hospitals such as Regional Referral Hospital and National Referral Hospitals. This is because these hospitals are semi-autonomous, self-accounting which means they can purchase what they deem necessary for their operation [29]. Furthermore, the findings cannot be generalised to private health facilities that may be buying acetic acid and provide training for their staff to provide cervical cancer screening using the VIA methods. While the survey was conducted in Acholi sub-region, Northern Uganda and the findings may not be generalised to other geographic areas in Uganda, it is important to note that staffing structures are uniform in public HCs across Uganda as approved by Ministry of Health [30]. This is because Uganda healthcare is delivered under the minimum health care package (MHCP) with prescribed uniform service standards based on the level of public health facility [29, 60]. Potential for bias as a result of opportunity sampling was mitigated by revisiting those HCs in which staff were absent on two further occasions following our first visit to seek to gather responses from the small number of staff who had not been available. To minimise potential for contamination of the data, we ensured that participants submit the questionnaires immediately after completion and requested that they did not discuss the content with their colleagues. There were only five staff members across the sites who did not complete the study questionnaires. There were also some HCs with only a small number of respondents; in two HCs III we recruited only one participant from each HC. Given that the HCs are managed centrally by DHO with uniform service standards, we do not believe this would have impacted the study findings.

## Conclusions

Our research demonstrates that there is a structural problem associated with providing cervical cancer screening services in HCs located in Northern Uganda which the Strategic Plan has not addressed. There is a need for training health workers and equipping the health facilities with equipment, resources, and reagents. This will enable the trained health workers to provide cervical cancer screening at health facilities. Improving staffing levels at the HCs will ensure that there are sufficient staff to provide cervical cancer screening services and will not suffer from burnout as result of being engaged in provision of other healthcare services. We observed that there is need for rooms for screening to ensure privacy of the women during screening service. Furthermore, there is a need to provide funding and transport to health facilities to facilitate cervical cancer activities in the community. Also, there is a need for continuous support and supervision of the health workers who are performing the cervical cancer screening services. It is important that absenteeism among health workers from HC III is addressed to ensure staff are available for the provision of cervical cancer screening services so that women who turn up for screening can be screened. Furthermore, to guide the activities of health workers, we recommend that both the District Local Governments and the Uganda Ministry of Health disseminate the Strategic Plan for Cervical Cancer Prevention and Control to the HCs. Addressing the structural barriers identified should be incorporated into the long-term plan for improving cervical cancer screening services provided by health workers in this region. This will enable Uganda government to attained the 90-70-90 WHO target by 2030 for a country to be on course for cervical cancer elimination as public health problem [20].

## Supplementary Information

**Additional file 1.** Survey Questionnaire for nurses/midwives/clinical officers.

## Data Availability

The dataset used for the present study’s conclusion can be accessible from the corresponding author on reasonable request if there is need.

## Abbreviations

AOR: Adjusted Odds Ratio
CC: Cervical cancer
COR: Crude Odds Ratio
DHO: District Health Officer
GAVI: Global alliance for vaccine and immunization
GU: Gulu university
HC: Health centre
HIV: Human immunodeficiency virus
LMICs: Low- and middle-income countries
MHCP: Minimum health care package
NGOs: Non-governmental organisations
OR: Odds Ratio
PI: Principal investigator
UNSW: University of New South Wales
VHTs: Village health teams
VIA: Visual inspection with acetic acid
95 % CI: 95 % Confidence interval
**χ**^**2**^: Chi Square
