# Supplementary material for: Provision of cervical cancer prevention services in Northern Uganda: a survey of health workers from rural health centres
Source: BMC Health Serv Res. 2021 Aug 11;21:794. doi: 10.1186/s12913-021-06795-5 (PMC8359606; doi:10.1186/s12913-021-06795-5)
Supplement: Supplementary file 1 — Additional file 1. Survey Questionnaire for nurses/midwives/clinical officers. [file 12913_2021_6795_MOESM1_ESM.docx]

**Survey Questionnaire for nurses/midwives/clinical officers**

**SECTION 1:**

Participant Code: ____/____/____/ Health facility Number: ____/____/

Health facility name…………………………………Parish name……………………………

Sub-county name……………………………...District name…………………..................…

Date: ____/____/_____. Interviewer’s Name……………………………

***Please mark a tick in the box applicable to your most appropriate response unless prompted for multiple responses.***

**SECTION 2: Questions on demographic and health centre characteristics (*One alternative*)**

| **S/No** | **Questions** | **Responses** |
| --- | --- | --- |
| 2.1 | How old are you now? | Completed Years |
| 2.2 | Gender | Male1  Female 2 |
| 2.3 | What is your current qualification? | Enrolled Nurse 1  Registered Nurse 2  Enrolled Midwife 3  Registered Midwife 4  Enrolled Comprehensive Nurse 5  Registered Comprehensive Nurse 6  Registered Bachelor of Science Midwifery 7  Registered Bachelor of Science Nursing 8  Registered Public Health Nurse 9  Clinical Officer 10 |
| 2.4 | How many years have you been working? | Completed Years |
| 2.5 | Have you ever been trained on how to conduct cervical cancer screening? | Yes 1  No 2 |
| 2.6 | Are there organization that the health centre partner with to provide cervical cancer screening to women? | Yes 1  No 2 |
| 2.7 | Does the health centre have any guideline for cervical cancer screening? | Yes 1  No 2 |
| 2.8 | Does the health facility receive fund for cervical cancer activities? | Yes 1  No 2 |

| **SECTION 3: Cervical cancer prevention services offered by the health centre.** | | |
| --- | --- | --- |
| **S/No** | **Questions** | **Responses** |
| 3.1 | Do you conduct cervical cancer screening in this health facility?  **If No 3.11** | Yes 1  No 2 |
| 3.2 | If yes in 3.1 above, are women counsel before being screened for cervical cancer? | Yes 1  No 2 |
| 3.3 | If yes in 3.1, what method do you use to screen for cervical cancer?  ***(Multiple responses allowed)*** | VIA 1  VILI 2  Pap smears 3  Liquid-based cytology 4  HPV DNA testing 5 |
| 3.4 | Are the community aware that the health facility offers cervical cancer screening? | Yes 1  No 2 |
| 3.5 | How often do you screen women for cervical cancer in this health facility? | 7 days a week 1  Monday to Friday 2  Three times in a week 3  Two times in a week 4  Once a week 5  Outreach by others 6 |
| 3.6 | If a woman screened have precancerous lesion, what do you offer her? If respond is  **“either 2 or 3” 3.8** | Refer her for treatment 1  Treat her from this facility 2  Tell her and do-nothing 3 |
| 3.7 | If respond in 3.6 is refer her for treatment, where do you refer her normally? | H/C IV 1  St. Joseph’s Hospital Kitgum 2  Kitgum Government Hospital 3  Ambrosoli Memorial Hospital 4  Gulu Regional Referral Hospital 5  St. Mary’s Hospital Lacor 6  Others, specify……………………….. 7 |
| 3.8 | Do you receive support supervision for cervical cancer prevention services offered in this health facility? **No 3.11** | Yes 1  No 2 |
| 3.9 | If yes in 3.8 above, name all the organizations providing support supervision for cervical. | ……………………………………………………  ………………………………………………….. |
| 3.10 | If yes in 3.8 above, how often do you receive support supervision? | Monthly 1  Every quarter 2  After every six months 3  Once a year 4 |
| 3.11 | If no in 3.1 above, why is cervical cancer screening not being conduct in this health facility? (***Multiple responses allowed)*** | No equipment/consumables 1  Lack of personnel 2  No space/room for screening 3  No one is trained to conduct  screening 4  No time for screening because  of heavy workload 5  Screening increases my workload 6  We are not interested 7  We luck skills to conduct screening 8  Others, specify………………………. 9 |
| 3.12 | Do you conduct HPV vaccination in this health facility?  **If No 3.14** | Yes 1  No 2 |
| 3.13 | Are the community members aware that the health facility provides vaccination against HPV infection? | Yes 1  No 2 |
| 3.14 | Do you provide health education on cervical cancer at the health facility?  **If Yes 3.16** | Yes 1  No 2 |
| 3.15 | If no in 3.14, why are health education not conducted in this health facility?  ***(Multiple responses allowed)*** | No health education material 1  Cervical cancer not a priority  disease here 2  Staffs have no interest 3  Government is not promoting 4  Others, specify………………………...5 |
| 3.16 | Does this health facility have information education & communication (IEC) material about cervical cancer? | Yes 1  No 2 |
| 3.17 | Do you conduct outreach health education in the community for cervical cancer?  **If Yes Stop here** | Yes 1  No 2 |
| 3.18 | If respond is no in 3.17 above, why?  ***(Multiple responses allowed)*** | No transport 1  No fund 2  No staff 3  No time because of heavy workload 4  Others, specify………………………..5 |
| 3.19 | Are you aware of the strategic plan for cervical cancer prevention and control in Uganda? | Yes 1  No 2 |
| 3.20 | If respond in 3.19 is yes, which period does the above strategic plan for cervical cancer prevention and control in Uganda covers? | 2010 – 2014 1  2015 – 2020 2  2016 – 2021 3  2017 – 2022 4 |
| 3.21 | Are you aware of the age group for cervical cancer screening using VIA? | Yes 1  No 2 |

**SECTION 4: Attitudes**

| **S/No** | **Questions** | **Responses** |
| --- | --- | --- |
| 4.1 | If you were trained to conduct cervical cancer, do you feel competent enough to conduct cervical cancer screening? | Yes 1  No 2 |
| 4.2 | Would you like to be trained on how conduct cervical cancer screening using VIA? | Yes 1  No 2 |
| 4.3 | Would you be willing to start cervical cancer screening in this health facility? | Yes 1  No 2 |
| 4.4 | If no in 4.3 above, could you tell us why you are not willing to start cervical cancer screening?  (Multiple responses allowed) | Will increase my workload 1  No support from supervisors 2  Government not interested 3  No equipment/consumable 4  No guideline/SoP 5  No space/room for screening 6  Others, specify……………………… 7 |

**END**

**For use by research assistant and PI only**

**Result of Questionnaire:** 1**.** Completed 2. Refused 3. Partly Completed

**PI Verification:** 1. Yes 2. No
